# 2064 Regulation of retinal protein O-GlcNAcylation by angiotensin-(1-7) and cAMP

**DOI:** 10.1017/cts.2018.114

**Published:** 2018-11-21

**Authors:** Sadie Dierschke, Amy Arnold, Michael M. Dennis

**Affiliations:** 1 Department of Neural & Behavioral Sciences, Penn State College of Medicine, Hershey, PA, USA; 2 Department of Cellular & Molecular Physiology, Penn State College of Medicine, Hershey, PA, USA

## Abstract

OBJECTIVES/SPECIFIC AIMS: Increased retinal protein O-GlcNAcylation occurs in response to hyperglycemia and contributes to diabetic retinopathy. Renin-angiotensin system (RAS) blockers reduce the incidence of diabetic retinopathy. Beneficial effects of RAS blockers are often attributed to production of angiotensin-(1-7) (Ang1-7). The objective here is to determine the impact of Ang1-7 on retinal protein O-GlcNAcylation. METHODS/STUDY POPULATION: C57/BL6 mice were fed a high-fat diet for 8 weeks and then treated for 3 weeks with either a vehicle control, the RAS blocker captopril, or captopril and the Ang1-7 receptor antagonist A779. R28 cells were used to assess levels of O-GlcNAcylated proteins in response to Ang1-7, and the role of cAMP was investigated with addition of forskolin, 6-Bnz-cAMP-AM, and 8-pCPT-2-O-Me-cAMP-AM to cell culture medium. RESULTS/ANTICIPATED RESULTS: Captopril attenuated retinal protein O-GlcNAcylation in mice fed a high-fat diet. This effect was reversed by A779. Ang1-7 attenuated protein O-GlcNAcylation and increased cAMP levels. Forskolin and the EPAC selective cAMP analog 8-pCPT-2-O-Me-cAMP-AM, but not the PKA selective cAMP analog 6-Bnz-cAMP-AM, attenuated O-GlcNAcylation. Inhibiting EPAC blocked the effect of forskolin, whereas inhibiting PKA did not. DISCUSSION/SIGNIFICANCE OF IMPACT: This study demonstrates a novel role for Ang1-7 in the retina and identifies a potential EPAC-dependent mechanism that regulates protein O-GlcNAcylation. Thus, future therapeutics targeted at an Ang1-7/EPAC axis in retina may be used to address DR.

